# Comparison of the main pathogenic microorganisms of various common oral diseases in children and adults

**DOI:** 10.1002/pdi3.35

**Published:** 2023-10-05

**Authors:** Siqi Zhou, Tong-Chuan He, Yuxin Zhang, Hongmei Zhang

**Affiliations:** 1 Chongqing Key Laboratory for Oral Diseases and Biomedical Sciences, The Affiliated Hospital of Stomatology, Chongqing Medical University, Chongqing, China; 2 Department of Pediatric Dentistry, The Affiliated Hospital of Stomatology, Chongqing Medical University, Chongqing, China; 3 Molecular Oncology Laboratory, Department of Orthopaedic Surgery and Rehabilitation Medicine, The University of Chicago Medical Center, Chicago, Illinois, USA

**Keywords:** adults and children, common oral diseases, oral microbiota

## Abstract

The microorganisms in the human body gradually change and maintain a dynamic balance with the development of physiology and pathology. Oral microbiota is one of the most important microbiota in human body. It is not only closely related to the occurrence and development of oral diseases, but also plays an important role in the overall health. In childhood, the population of oral microorganisms is relatively small, but with the growth of age and tooth development, the species and quantity of oral microorganisms are gradually increasing. Different oral diseases also have their corresponding main microorganisms, and these dominant microorganisms change at different stages of the disease. In this review, we summarized and compared the main pathogenic microorganisms of several common oral diseases in children and adults. In addition, the possible association and difference between adults and children of the main pathogenic microorganisms in different stages of the same or different diseases are also discussed in order to provide research data for the development and diagnosis of common oral diseases in children and adults.

## 1 | INTRODUCTION

Since the birth of each newborn, bacterial communities begin to colonize the human body.^1,2^ A large number of previous studies have shown that the colonization of neonatal oral flora comes from the vertical transmission of their mothers, horizontal transmission between individuals, transmission in the process of diet and feeding, and contact with the environment.^2–5^ However, the microbiota in the human body is not immutable and frozen; with the change of dentition and reconstruction of oral function, as one of the most diverse environments in our body, the number, type, and even dominant bacteria of oral flora will change accordingly.^1,5–8^ At the beginning, in edentulous stage, the main location of oral flora is at the surface of oral mucosa and^3^ the main bacteria are gram-positive facultative bacteria, followed by gram-negative anaerobic bacteria.^6,9^ *Streptococcus salivarius* is the most commonly isolated bacteria in the oral mucosa of newborn infants because it has better adhesion with buccal mucosa and tongue^1^; it keeps a relatively stable growth in the first 3 months of the newborn but begins to decrease after 3 months, which is believed to be related to the eruption of the first deciduous tooth in the mouth of children.^1,10^ At the same time, *Streptococcus mutans* begin to accelerate its colonization, and it is due to the greater adhesion of *S. mutans* to the tooth surface compared with other bacteria in the mouth.^8^ The proportion of *S. mutans*, Pasteurellaceae, and Enterobacteriaceae in primary dentition stage is higher than that in other dentition stages.^8^ After the eruption of the last deciduous tooth, the operational taxonomic units of bacteria that can be detected in the mouth have reached 550.^4^ The proportion of Pasteurellaceae and Carnobacteriaceae in mixed dentition stage is higher.^8^ In the period of permanent dentition, there are 10,000 microbial system types in dental plaque alone, which is far more than the more than 700 oral microbial system types detected by traditional methods.^11^

Mostly, it is worth noting that fungi and viruses are also present in the oral cavity of a newborn baby after birth, and these fungi and viruses may also be occasional causative factors in certain contexts, such as congenital immunodeficiency. Candida is the most frequently detected fungus; other fungi such as *Cladosporium*, *Aureobasidium*, *Saccharomycetales*, *Aspergillus*, *Fusarium*, and *Cryptococcus* also exist at the same time.^12–14^ At present, there is not enough research on the viruses colonized in the mouth of newborns under physiological conditions, mainly focusing on the viruses under pathological conditions, including HIV, herpes simplex virus (including HSV-1 and HSV-2), and coxsackie virus.^15–20^

Therefore, the oral microbiota changes with time and maintains a dynamic balance. The oral microbiota in different periods is different, which also conforms to the view of ecological succession.^6,21^ Understanding the differences and changes of oral microbiota has long been considered as a very important aspect for disease prediction and diagnosis. However, nowadays, research on the main microorganisms of common oral diseases in children and adults mainly focuses on the comparison of oral flora between parents and children with the same disease or the influence of one party's illness on the other party's oral flora or other physical conditions. This article believes that by comparing the main microorganisms of common oral diseases in children and adults, new discoveries may be made about the understanding of the same disease in different age groups, and it is also of profound significance to prevent common oral diseases in children and adults.

## 2 | COMPARISON OF THE MAIN MICROBIOTA BETWEEN CHILDREN AND ADULTS IN COMMON ORAL DISEASES

### 2.1 | Caries

Caries is considered to be a common oral disease for children and adults, and it can be regarded as one of the most popular diseases in the world.^22–24^ This is a common dental hard tissue defect disease caused by bacteria, food, host, and time.^25^ Previous cross-sectional studies have shown that there are core biogroups in caries-related bacteria, and higher caries prevalence is related to lower bacterial diversity. With the severity of caries, microbial diversity gradually decreases.^26–29^ According to the detection of oral flora of adults with dental caries, the core flora of adult plaque microbiota includes *S. salivarius*, Veronicus, Leptotrichia, *Streptococcus parasanguinis*, Capnocytophaga, Prevotella, Actinomyces, Corynebacterium, Neisseria, Rothia, and Megasphaera.^27,29^ In addition, some uncommon bacteria were also detected with increased abundance, including Atopobium, Cryptobacterium, Lactobacillus, Mogibacterium, Ochrobactrum, Pseudomonas, Rhizobium, Alloprevotella, Bacteroides, Centipeda, Campylobacter, Megasphaera, Mycoplasma, Gammaproteobacteria, Cardiobacterium, and Pasteurellaceae (Table 1), and they are considered as potential cariogenic bacteria.^27,30,31^

Surprisingly, *S. mutans* do not play a dominant role in adult caries but plays an important role in children's caries. Majda Dzidic et al. compared the bacteria in saliva of children with caries and children without caries from 3 months to 7 years old by high-throughput sequencing. The experimental results showed that after 2 years of age, caries affected and caries-free children had significantly different bacterial colonization patterns, but before 2 years of age, the difference was not obvious, and before 7 years of age, the incidence rate of caries was not significantly related to bacterial diversity or bacterial abundance. After 7 years of age, compared with children without dental caries, children with dental caries have higher levels of *S. mutans* and *Streptococcus cristatus* and lower level of *Streptococcus dentisani*.^5^ Jørn A. Aas et al. believed that there were more kinds of health-related bacteria isolated from the plaque of permanent teeth than that of deciduous teeth, but the main bacteria in deciduous teeth were still included in the plaque of permanent teeth. As for pathogenic bacteria, compared with deciduous teeth, there is a significantly greater overall diversity of bacteria related to permanent teeth caries. The distribution of bacteria at the beginning of caries (i.e., intact enamel and white plaque on enamel surface) is also more obvious and complex than that in the identified caries. At the same time, the main bacteria of adults and children are different in different stages and parts of caries development. When the caries was confined to the enamel and superficial dentin, high levels of *S. parasanguinis* and *S. salivarius* were observed in the dental plaque of both permanent and deciduous teeth, but high levels of *Corynebacterium* sp. clone AK153, and *Actinomyces gerencseriae* were detected in deciduous dentition and high levels of *Leptotrichia* sp. clone GT018, *Campylobacter gracilis*, and *Selenomonas* sp. clone EY04 were detected in permanent dentition. When the caries progressed to the deep dentine layer, the microbial community was dominated by *S. mutans*, *Lactobacillus* spp., *Propionibacterium* sp. strain FMA5, *Atopobium genomospecies* C1 (permanent teeth), and *Bifidobacterium* spp. (deciduous teeth).^32^

In addition, it should not be ignored that early childhood caries (ECC) is a kind of caries that develops rapidly and has a wide range, especially in children under 6 years of age. The bacteria contained in the oral cavity of young children with ECC may be different from pathogenic bacteria of adult caries. The study found that children with severe early childhood caries had elevated levels of *streptococci* (especially *Streptococcus mutans*), *Leptosella, Prevoella*, oral bacillus, bifidobacteria, *Porphyromonas* and *Salmonella* in the mouth compared with children without dental caries.^33^ Kanasi et al. compared the bacteria at different positions in oral cavity of children with EEC and caries-free children and concluded that the related species of EEC mainly include: *S. mutans*, *S. mutans* combined with *Streptococcus sobrinus*, *Lactobacillus vaginalis*, *Staphylococcus intermedius*, *Lactobacillus fermentum*, *Lactobacillus gasseri*, *Actinomyces odontolyticus*, *Actinomyces israelii*, and *Veillonella parvula*. Among them, the pathogenic rate of *S. mutans* combined with *S. sobrinus* to EEC is higher than that of single *S. mutans*, and among the EEC detected by non *S. mutans*, it is *S. intermedius* that is more relevant. *Prevotella nigrescens* is the only species that is significantly associated with caries. Furthermore, the detected bacteria related to caries have a much higher content in dental plaque than in tongue and saliva, but it has little relationship with the tooth position.^34^ Combined with other studies, it is found that *S. mutans* and Lactobacillus are the main bacteria in EEC, especially *S. mutans*, whose high number is an obvious feature of caries in young children.^35–40^ The role of *S. mutans* in the development of caries may be related to its characteristics of acid production and acid resistance, and it can provide biofilm matrix for other cariogenic bacteria when the biofilm matures, thus, protecting the acidic environment where acid production and acid bacteria grow.^36^ Moreover, other microorganisms such as human herpesvirus type 4 (Epstein–Barr virus [EBV]) and *Candida albicans* were confirmed to have higher levels in EEC than subjects with common caries or without caries^38,41,42^ (Table 1).

Therefore, during the occurrence and development of caries, the pathogenic microorganisms of caries in adults and children are different. Adults mainly include salivary streptococcus, blood streptococcus, etc. *S. mutans* plays an important role in the development of caries in children. The microbial diversity of adults is higher than that of children.

### 2.2 | Pulpal disease

Pulp disease is a general term for dental pulp tissue diseases. This section mainly introduces the microorganisms in the common diseases of pulpitis and pulp necrosis caused by infection and is limited to root canal. Bacteria are the most important pathogenic factor of dental pulp disease, mainly including facultative anaerobes and obligate anaerobes. Due to the characteristics of tooth structure, bacterial infection mainly comes from tooth defect, periodontal infection, and blood infection, among which microorganisms play a vital role. Many studies have shown that significant changes have taken place in the microbiota in the root canal of dental pulp infection, and the richness of dominant bacteria and species in different infection stages are also different.^43,44^ Dental caries is an important cause of pulp infection. In the severe caries stage, when the decay is very close to the pulp, bacteria can infect the pulp through the dentinal tubules.^45^ With the further development of the disease, the symptoms of reducible pulpitis, irreducible pulpitis, and pulp necrosis appear immediately.

With the development of caries, actinomycetes decreases and chlamydia increases.^44^ As far as adults are concerned, Lactobacillus, Pseudoramibacter, Streptococcus, Propionibacterium, Olsenella, Actinomyces, Capnocytophaga, Capnocytophaga, and Leptotrichia are the main bacteria of the reducible pulpitis.^46,47^ Irreducible pulpitis bacteria mainly include Lactobacillus, Streptococcus, Prevotella, Veron, Corynebacterium, Cutibacterium, and Porphyromonas.^44,48^ Pulp necrosis bacteria mainly include Prevotella, Porphyromonas, and Peptostreptococcus. The relative abundance of Lactobacillus in irreducible pulpitis is higher than that in reducible pulpitis.^44^

For children with irreducible pulpitis, root canal has been detected to be rich in *Enterococcus faecalis*, *Escherichia coli*, *S. mutans*, *Staphylococcus aureus*, and *C. albicans*. The content of anaerobes is less, and facultative anaerobes are more dominant than strict anaerobes.^49^ The main bacteria detected in deciduous teeth with pulp necrosis include *E. faecalis*, *S. salivarius*, Hemolytic Streptococci (*α*, *β*, and *γ*), *E. coli*, *S. aureus*, and *Proteus mirabilis*.^50–52^ Similarly, they are dominated by facultative anaerobes (Table 2). However, at present, the research objects of dental pulp infection bacteria mainly focus on individuals suffering from periapical infection, especially deciduous teeth pulpitis. Due to the difficulty in obtaining materials, the relevant research results are even rarer. Therefore, for the infection that has not developed to the stage of periapical periodontitis, more research is needed to detect the dominant bacteria and community characteristics.

### 2.3 | Periapical periodontitis

With further development of the lesion, microorganisms in the necrotic pulp may spread to and around the root apex through special structures such as apical foramen, dentinal tubules, lateral root canals, apical furcations, and accessory root canals, leading to periapical periapical Inflammation, and even periapical granuloma, apical cyst or apical abscess may occur, eventually leading to tooth pain, looseness, formation of mucosa or buccal fistula and other symptoms.^53,54^ For nonstandard root canal therapy or root canal therapy failure, periapical periodontitis is often one of the most common sequelae. However, deciduous teeth with periapical periodontitis may have some impact on the development or eruption of subsequent permanent teeth, such as the occurrence of Turner teeth.^55,56^ The bacteria in periapical periodontitis are slightly different from those in pulpitis.

The most common microorganisms isolated from adult periapical periodontitis are mainly anaerobic bacteria. Wayman et al. successfully isolated *Staphylococcus epidermidis*, *Fusobacterium nucleatum*, *Propionibacterium acnes*, *Peptostreptococcus micros*, and *Bacteroides gracilis* from periapical tissues.^57^ Combined with other studies, it can be concluded that the bacteria of adult periapical periodontitis are mainly Actinomycetes, Fusobacterium, and Prevotella, including *C. gracilis*, *Porphyromonas endodontalis*, *Bacteroides forsythus*, *P. acnes*, *Capnocytophagagingivalis*, *F.nucleatum* ssp. *nucleatum*, *F. nucleatum* ssp. *polymorphum*, *Prevotella intermedia*, *Treponema denticola*, *Streptococcus constellatus*, *Actinomyces naeslundii*, *Actinobacillus actinomycetemcomitans (Aa)*, *Eubacterium sabureum*, *P. micros*, *V. parvula*, *Streptococcus anginosus*, *Leptotrichia buccalis*, *Capnocytophaga sputigena*, *Streptococcus gordonii*, *Actinomyces viscosus*, etc.^58,59^ There are also a few research results showing that the root canals or periapical tissues of teeth with persistent infection of periapical periodontitis have also been found to contain fungi, especially *C. albicans*, in addition to *Candida glabrata*, *Candida unspicua*, *Geotricum candium*, etc.^60^ EBV and human cytomegalovirus (HCMV) were also found in periapical tissues,^61,62^ which suggests that viruses may also play a role in the occurrence and development of periapical periodontitis.

In children's periapical periodontitis, the detection rates of Porphyromonas, *T. denticola*, *E. faecalis*, Prevotella, Fusobacterium, Peptostreptococcus, Streptococcus, Eubacterium, Lactobacillus, Campylobacter, and Bulleidia were higher. Among them, Prevotella is the most dominant bacteria in periapical abscess of deciduous teeth, followed by Fusobacterium, Digestive Streptococcus, and Porphyromonas.^63–65^ Teuta Kutllovci et al. found that the dominant bacteria group of periapical infection in children is anaerobic bacteria, mainly including *Actinomyces mayeri*, *A. naeslundi*, *Actinomyces odontoliticus*, Fusobacterium, Veillonella, and Lactobacillus. In acute infection, the detection rate of Actinomycetes in deciduous teeth is higher than that in chronic infection group, while the proportion of Fusobacterium in deciduous teeth is not closely related to the acute and chronic stages, and its content is always higher than that in permanent teeth group (Table 3).^65^

### 2.4 | Periodontal disease

The most common forms of human periodontal disease are gingivitis and periodontitis. Gingivitis is an inflammatory disease that is limited to gingival tissue without causing attachment loss and alveolar bone absorption and is reversible. The bacterial flora related to adult gingivitis mainly includes TM7, *Leptotrichia*, *Selenomonas*, *Streptococcus*, *Veillonella*, *Prevotella*, *Neisseria* spp., *Hafnia alvei*, *C. albicans*, *Lautropia*, *Pseudomonas aeruginosa*, and *Haemophilus*.^66,67^

Periodontitis is a chronic inflammatory disease. Its main symptoms include gingival redness and bleeding, alveolar bone absorption, and tooth mobility. The damage to tissues caused by periodontitis is irreversible. Its main cause is the presence of specific bacteria in the subgingival plaque biofilm and the change of the flora.^68,69^ For this reason, it is of great significance to determine the dominant flora of periodontitis for the understanding and treatment of this disease. So far, *Aggregatibacter actinomycetem*, *Tannerella forsythensis*, and *Porphyromonas gingivalis* are almost recognized as the main pathogens leading to the onset and development of periodontitis.^70,71^ The dominant bacteria in the saliva of patients with periodontitis are *P. gingivalis*, *Ac. actinomycetemcomitans*, and *S. mutans*.^29,72,73^ Tanner et al. compared the flora on the back of the tongue and subgingival of early adult periodontitis samples and found that *T. denticola*, *Filifactor alocis*, *F. nucleatum*, and *P. gingivalis* were related to progressive periodontitis, and the subgingival samples were more reliable than tongue samples when detecting periodontal pathogens.^74^ In addition, Riviere et al. found that the abundance of *P. gingivalis* under the gingiva of patients with early periodontitis was higher than that of healthy people, and the level of *P. gingivalis* in patients with gingivitis was higher than that in patients with early periodontitis.^75^ Colombo et al. found that subgingival flora of adult periodontitis patients had more kinds and quantities of pathogenic bacteria than that of healthy individuals, such as *Parvimonas micra*, *C. gracilis*, *Tannerella forsythia*, *P. gingivalis*, *Megasphaera micronuciformis*, *Eubacterium nodatum*, *Selenomonas noxia*, *Leptotrichia*, *V. parvula*, *Prevotella* spp., *Treponema* spp., *Eikenella corrodens*, *Pseudoramibacter alactolyticus*, TM7 spp. oral taxon OT 346/356, *Bacteroidetes* sp. OT 272/274, *Solobacterium moorei*, *Desulfobulbus* sp. OT 041, *Brevundimonas diminuta*, *Sphaerocytophaga* sp. OT 337, *Shuttleworthia satelles*, *F. alocis*, *Granulicatella adiacens*, *Mogibacterium timidum*, *Veillonella atypica*, and *Mycoplasma salivarium*.^76–78^ It has also been proved that the bacteria related to periodontal injury are mainly enterobacteria and *C. albicans*, *Neisseria* spp., *P. aeruginosa*, *Olsenella uli*, *Hafnia alwei*, *Serratia marcescens*, and *F. alocis*.^67^

However, different from most oral anaerobes, with the growth of age, some periodontal pathogens usually do not colonize the oral cavity in the early stage, and most healthy children do not suffer from periodontitis.^79,80^ Therefore, the bacteriological study of periodontal disease in healthy children is still rare. Morinushi et al. found that *P. gingivalis* and Actinomycetes appeared as early as 3 years old in children, and their existence was related to the onset and severity of gingivitis in children.^81^ Sheila Cavalca Cortelli et al. detected that *P. gingivalis* was the most abundant bacteria in the gingival biofilm samples of 196 children with gingivitis, followed by *Tannerella forsythensis*, *P. intermedia*, *Aggregatibacter actinomycetemcomitans*, and *Campylobacter rectum*.^82^ *P. intermedia*, *Eikenella corrodes*, Black-pigmented Bacteroides species, *Bacteroides intermedius*, *Ac. actinomycetemcomitans*, *F. nucleatum*, etc. also account for a high proportion of children's gingivitis bacteria.^83^ In addition, Moore et al. found that *F. nucleatum*, Actinomyces WVa 963, Selenomonas D04, and *Treponema socranskii* were the main species related to the increase of gingival index scores in children and adults. Compared with adults who also suffer from gingivitis, the proportion of Leptotrichia species detected in children's dental plaque is 3 times higher, the proportion of Capnocytophaga species is 2.5 times higher, and the proportion of Selenomonas species is 2.3 times higher, but the proportion of Fusobacterium, Eubacterium, and Lactobacillus in adults is higher.^84^ As for children with invasive periodontitis, *Aa* is considered to be the main pathogenic bacteria in subgingival plaque, and the abundance of *F. alocis*, Tannerella, *S. moorei*, *Parvimonas microca*, and Capnocytophaga is also high.^85^

Researchers analyzed the viruses detected in subgingival samples of patients with periodontitis and found that the levels of EBV, HCMV, and HSV-1 increased with the severity of periodontal disease,^86^ and the amounts of these viruses were related to *P. gingivalis, T. denticola, Fusobacterium periodonticum*, and *S. aureus* in periodontal tissue.^87^ However, there is no definite evidence that there must be a relationship between the virus and the pathogenic bacteria of periodontitis,^88–90^ perhaps this can also become a new research hotspot.

Moreover, we see that the main microorganisms of periodontitis or gingivitis in children and adults are similar, but the proportion of dominant microorganisms is different between adults and children (Table 4). At present, most of the microbiological samples related to periodontitis are taken from saliva or subgingival plaque. There are relatively few studies on gingival crevicular fluid. Although there are quite rich studies on adult periodontitis, there are few studies on children with periodontitis, so we need to use more samples to further explore the oral dominant microorganisms of children's periodontitis.

### 2.5 | Oral mucosal disease

Oral mucosal disease refers to the general term of oral mucosal and soft tissue diseases. The causes of mucosal disease are complex, and most of them are related to the general and mental state. This part mainly introduces the oral microorganisms related to denture stomatitis, recurrent aphthous ulcer, and angular stomatitis. The main microorganisms of adults and children in common mucosal diseases are summarized in Table 5.

#### 2.5.1 | Denture stomatitis

Denture stomatitis is a mucosal disease in the mouth of patients wearing dentures. It mainly occurs in and near the mucosa covered by dentures. It shows that the corresponding mucosa has edema, erythema, pseudomembrane, ulcer or even erosion, and the patient feels pain or burning.^91–93^ Elderly people who have lost their teeth and children who have early occlusion induction are the two main groups who use dentures. Among them, the incidence rate of denture stomatitis is also high.^92^ Due to poor oral hygiene or inadequate denture cleaning, biofilms quickly form on the denture surface. These biofilms are composed of candida and other bacteria, which can not only adhere to the denture surface, but also gradually spread to other parts of the mouth. So far, there are many evidences that *C. albicans* infection is an important factor leading to the disease,^94–96^ but due to the diversity of oral bacterial community, more and more studies have found that there are many other kinds of microorganisms related to denture stomatitis. In healthy adults with denture stomatitis, the main microorganisms detected include *C. glabrata*, *Candida tropicalis*, *Candida krusei*, *Candida parapsilosis*, *Candida dubliniensis*, *S. aureus*, *S. epidermidis*, *Klebsiella pneumoniae*, *P. aeruginosa*, and *Pseudomonas fluorescens*.^97,98^ Webb et al. found that the location of candida colonization is mainly on the surface of dentures. Compared with denture wearers without denture stomatitis, denture plaque is composed of basically similar flora, but the contents of *Streptococcus* species, *Actinomyces* species, and *Veillonella* species in denture stomatitis patients are less, and the content of *Propionibacterium* species and *Lactobacillus* species is higher.^95^

In most cases, the dentures worn by children are orthodontic movable appliances. Due to the long wearing time, children's self-oral cleaning is not in place and other reasons are that the children wearing dentures also often have symptoms such as swollen mucosa. Many studies have found that the number of *S. mutans*, *E. coli*, *S. aureus*, *P. aeruginosa*, Candida, and Lactobacillus is higher in children who are treated with movable orthodontic appliances, and the content of Streptococcus is significantly higher than that of Candida.^99–102^ There is a direct relationship between the use of removable orthodontic appliances and the increase of periodontal pathogens.

#### 2.5.2 | Recurrent aphthous ulcer

Recurrent aphthous ulcer is a recurrent, self-limited oral mucosal disease with complex etiology. The clinical symptoms are red or yellowish depressions on the oral mucosa, accompanied by erosion and pain. The disease usually first occurs in children or teenagers and often persists throughout life.^103–105^ At present, the research on recurrent aphthous ulceration (RAU) related pathogens is still in a small stage, but few people believe that Streptococcus, especially *S. sanguis* may participate in the occurrence of RAU, and RAU may be a result of T cell-mediated reaction to *S. sanguis*.^106,107^ Some subpopulations of Prevotella, Neisseria, and Streptococcus, such as *Prevotella melaninogenicus*, *Neisseria lactamica*, *Neisseria mucosa*, *Neisseria saprophaga*, *Neisseria subflava*, *Streptococcus viridans*, *S. mutans*, and *S. aureus*, may be related to the occurrence of RAU.^108^ The significant increase in the number of Prevotella at the ulcer site more effectively indicates that Prevotella is closely related to RAU.^109^ For *Helicobacter pylori*, there is still no direct evidence to prove that it is related to the occurrence and development of RAU.^110^ There is less research on viruses. At present, only a small number of people think that EBV is related to the epithelial cells of recurrent aphthous stomatitis before ulcer.^111^

#### 2.5.3 | Angular stomatitis

Angular stomatitis is an infectious disease that occurs at the joint of the upper and lower lips, mainly manifested as chaps, flushing, dander or erosion. The bacteria associated with adult angular stomatitis are mainly found to be *Candida albieans*, *C. parapsilosis*, *C. krusei*, *S. aureus*, Beta aerolytic streptococci, *E. coli*, and other microorganisms.^112,113^ Microbes related to children's angular stomatitis mainly include *S. aureus*, *Enterobacter cloacae*, *Staphylococcus warneri*, *C. tropicalis*, *Dermacoccus nishinomiyaensis*, etc. *S. aureus* is the most dominant bacteria.^114^ *K. pneumoniae*, *S. gordonii*, *Streptococcus oralis*, *Bordetella bronchiseptica*, *Streptococcus pseudoporcinus*, *Streptococcus agalactiae*, *S. salivarius*, *Gamella morbilorium*, and other bacteria are also suspected to be related to the progress of angular stomatitis.^114^

## 3 | CONCLUSION

Due to the difficulty of plaque collection in clinical practice, the main microbial test samples are mainly from saliva, tongue back, dental plaque, or affected tissues. With the progress of science and technology, the identification of microorganisms has gradually changed from isolation and culture of microorganisms to DNA finger-printing, and then to the current high-throughput sequencing or mass spectrometry identification technology. The number of microorganisms detected in the mouth has increased from more than 700 to more than 10,000. Our understanding of microorganisms has also changed from simple morphology and classification to current gene, protein, or metabonomics. The relationship between microorganisms or between microorganisms and hosts is gradually becoming clear. However, there are many kinds and large quantities of microorganisms. The role of microorganisms in different environments and their impact on human diseases are still a major research focus today. In this review, the main microorganisms of adults and children in common oral diseases are collated and compared horizontally by consulting relevant literature, which may have new enlightenment for the microbial research of the same oral disease at different stages.

Therefore, we believe that the same disease has different microbial species types and abundance between adults and children, which may be related to the replacement of microbial populations during the growth and development of healthy individuals, or to the environmental changes, oral hygiene maintenance, or the mutual influence of other diseases during the growth process. First, at different stages of the same disease, the population richness and dominant species of microorganisms are different. For example, in the acute stage of periapical periodontitis of deciduous teeth, *A. mayeri* is the most abundant bacteria detected, and in the chronic stage, it becomes *Anaerococus prvoti*.^65^ With the development of dental caries, the number of actinomycetes decreases, and the increase of chlamydia is also a direct proof of this view.^44^ Secondly, the core microorganisms of the same disease are different between adults and children. Similarly, in acute periapical infection, even though the dominant bacteria in adults and children are *A. mayeri*, the relative number of these microorganisms detected in adults is higher than that in children. In the chronic phase, the relative number of these microorganisms detected in adults is basically lower than that in children. The dominant bacteria in adults is still *A. mayeri*, and in children it becomes *A. prvoti*.^65^ Thirdly, the main microbial species of children and adults identified in different diseases also have some overlap. For example, children with dental caries in young children more frequently detect subgingival species such as *P. micromera*, *F. alocis*, *P. gingivalis*, *Prevotella nigescens*, *Porphyromonas intantalis*, *Aa*, and *L. buccalis*. This may be related to the onset of gingivitis in children or even periodontitis in adolescence, and this part of the population may have a greater probability of periodontitis in adulthood.^34^ It is worth noting that the occurrence and development of these common oral diseases are not only caused by one or several microorganisms. Various microorganisms interact to form biofilms, thus, exerting their pathogenicity. The dominant microorganisms at different stages are also different, which is also affected by the original oral flora. Some bacteria were originally related to health, but due to the addition of other bacteria, it may become an opportunistic pathogen.

At present, the research on the main flora of oral diseases in adults and children is still mostly focused on the comparison of oral flora between mothers and children or the influence of mothers' illness on oral flora of children. In terms of longitudinal research, for example, there are still few studies on the main oral flora of patients with the same disease from childhood to adulthood, or on oral diseases with a high prevalence rate in the same family or region, oral flora changes of adults and children, etc. We can only summarize the main flora of children and adults with common and different oral diseases by comparing the existing research results and by trying to provide some new ideas or directions for this research.

## Figures and Tables

**TABLE 1:** The main pathogenic microorganisms of dental caries in adults and children.

Disease	Early childhood caries		Caries in children	Caries in adult	
Main microorganisms	*S. mutans* *S. sobrinus* *S. intermedius* *Lactobacillus vaginalis* *Lactobacillus fermentum* *Lactobacillus gasseri* *Actinomyces odontolyticus* *Actinomyces israelii*	*V. parvula* *Prevotella* Bifidobacterium Porphyromonas Salmonella Epstein–Barr virus *Candida albicans*	*S. mutans* *S. cristatus* *S. parasanguinis* *S. salivarius* Corynebacterium *A. gerencseriae* *Lactobacillus* spp. Propionibacterium *Bifidobacterium* spp.	*S. salivarius* Veillonella Leptotrichia *S. parasanguinis* Capnocytophaga Prevotella Actinomyces Corynebacterium Neisseria Rothia Pasteurellaceae	Megasphaera Atopobium Cryptobacterium Lactobacillus Mogibacterium Ochrobactrum Pseudomonas Campylobacter Mycoplasma Gammaproteobacteria

**TABLE 2:** The main pathogenic microorganisms of pulpal disease in adults and children.

Disease	Adult reversible pulpitis	Adult irreversible pulpitis	Adult pulp necrosis	Irreducible pulpitis in children	Pulp necrosis in children
Main microorganisms	Lactobacillus Pseudoramibacter Streptococcus Propionibacterium Olsenella Actinomyces Capnocytophaga Leptotrichia	Lactobacillus Streptococcus Prevotella Veillonella Corynebacterium Cutibacterium Porphyromonas	Prevotella Porphyromonas Peptostreptococcus	*Enterococcus faecalis* *Escherichia coli* *S. mutans* *Staphylococcus aureus* *Candida albicans*	*Enterococcus faecalis* *S. salivarius* *S. hemolytic* (α,β,γ) *Escherichia coli* *Staphylococcus aureus* *Proteus mirabilis*

**TABLE 3:** The main pathogenic microorganisms of periapical periodontitis in adults and children.

Disease	Adult periapical periodontitis		Periapical periodontitis in children
Main microorganisms	*Staphylococcus epidermidis* *Fusobacterium nucleatum* *Propionibacterium acnes* *Peptostreptococcus micros* *Bacteroides gracilis* *Campylobacter gracilis* *Porphyromonas endodontalis* *Bacteroides forsythus* *Propionibacterium acnes* *Capnocytophaga gingivalis* *Prevotella intermedia* *Treponema denticola* *Streptococcus constellatus*	Actinomyces *naeslundii* *Aa* *Eubacterium saburreum* *Peptostreptococcus micros* *Veillonella parvula* *Leptotrichia buccalis* *Capnocytophaga sputigena* *Streptococcus gordonii* *Actinomyces viscosus* *Candida albicans* *Candida glabrata* *C. unspicua* *Geotricum candium*	Porphyromonas *Treponema denticola* *Enterococcus faecalis* Prevotella Fusobacterium Peptostreptococcus Streptococcus Eubacterium Lactobacillus Campylobacter Bulleidia *Actinomyces mayeri* *Actinomyces naeslundi* *Actinomyces odontoliticus* Veillonella

Abbreviation: *Aa, Actinobacillus actinomycetemcomitans*.

**TABLE 4:** The main pathogenic microorganisms of periodontal disease in adults and children.

Disease	Adult gingivitis	Adult periodontitis		Gingivitis in children	Periodontitis in children
Main microorganisms	Leptotrichia Selenomonas Streptococcus Veillonella Prevotella *Neisseria* spp. *Hafnia alvei* *C. albicans* Lautropia *P. aeruginosa* Haemophilus	*Aggregatibacter actinomycetem* *Tannerella forsythensis* *Porphyromonas gingivalis* *S. mutans* *Treponema denticola* *F. alocis* *Fusobacterium nucleatum* *Parvimonas micra* *Campylobacter gracilis* *Tannerella forsythia* *Porphyromonas gingivalis* *Megasphaera micronuciformis* *Eubacterium nodatum* *Selenomonas noxia* Enterobacteria *C. albicans* *Neisseria* spp. *P. aeruginosa* *O. uli*	Leptotrichia *Veillonella parvula* *Prevotella* spp. *Eikenella corrodens* *Pseudoramibacter alactolyticus* *Solobacterium moorei* *Brevundimonas diminuta* *Shuttleworthia satelles* *Granulicatella adiacens* *Mogibacterium timidum* *Veillonella atypica* *Mycoplasma salivarium* *Hafnia alwei* *Serratia marcescens* *Filifactor alocis* EBV HCMV HSV-1	*Porphyromonas gingivalis* *Tannerella forsythensis* *Prevotella intermedia* *Aggregatibacter actinomycetemcomitans* *Campylobacter rectus* *Eikenella corrodens* Black-pigmented Bacteroides *Bacteroides intermedius* *Fusobacterium nucleatum* Selenomonas *Treponema socranskii* Leptotrichia Capnocytophaga	*Aa* *Filifactor alocis* Tannerella Solobacterium moorei *Parvimonas micra* Capnocytophaga EBV HCMV HSV-1

Abbreviations: *Aa, Actinobacillus actinomycetemcomitans;* HCMV, human cytomegalovirus.

**TABLE 5:** The main pathogenic microorganisms of oral mucosal disease in adults and children.

Disease	Adult denture stomatitis		Dental stomatitis in children	Adult angular stomatitis	Angular stomatitis in children
Main microorganisms	*C. albieans* *C. glabrata* *C. tropicalis* *C. krusei* *C. parapsilosis* *C. dubliniensis*	*S. aureus* *S. epidermidis* *Klebsiella pneumoniae* *P. aeruginosa* *P. fluorescens*	*S. mutans* *Escherichia coli* *S. aureus* *P. aeruginosa* *Lactobacillus*	*C. albieans* *C. parapsilosis* *C. krusei* *S. aureus* β-hemolytic streptococci *Escherichia coli*	*S. aureus* *Enterobacter cloacae* *Staphylococcus warneri* *C. tropicalis* *Dermacoccus nishinomiyaensis*

## Data Availability

Data sharing not applicable to this article as no datasets were generated or analyzed during the current study.
